# Parental experiences of caring for preterm infants in the neonatal intensive care unit, Limpopo Province: a descriptive qualitative study exploring the cultural determinants

**DOI:** 10.1186/s12913-024-11117-6

**Published:** 2024-05-28

**Authors:** Madimetja J. Nyaloko, Welma Lubbe, Salaminah S. Moloko-Phiri, Khumoetsile D. Shopo

**Affiliations:** NuMIQ Research Focus Area, North-West University, Potchefstroom, South Africa

**Keywords:** Parental experiences, Preterm infant, Neonatal intensive care unit, Descriptive, Cultural determinants, Healthcare professionals, Culturally sensitive care

## Abstract

**Background:**

Parent-infant interaction is highly recommended during the preterm infant hospitalisation period in the Neonatal Intensive Care Unit (NICU). Integrating culturally sensitive healthcare during hospitalisation of preterm infants is critical for positive health outcomes. However, there is still a paucity of evidence on parental experience regarding cultural practices that can be integrated into preterm infant care in the NICU. The study explored and described the cultural determinants of parents that can be integrated into the care of preterm infants in the NICU.

**Methods:**

A descriptive qualitative research design was followed where twenty (*n*=20) parents of preterm infants were purposively selected. The study was conducted in the NICU in Limpopo using in-depth individual interviews. Taguette software and a thematic analysis framework were used to analyse the data. The COREQ guidelines and checklist were employed to ensure reporting standardisation.

**Results:**

Four themes emerged from the thematic analysis: 1) Lived experienced by parents of preterm infants, 2) Interactions with healthcare professionals, 3) Cultural practices concerning preterm infant care, and 4) Indigenous healthcare practices for preterm infants.

**Conclusions:**

The study emphasised a need for healthcare professionals to understand the challenges parents of preterm infants face in NICU care. Furthermore, healthcare professionals should know indigenous healthcare practices to ensure relevant, culturally sensitive care.

**Supplementary Information:**

The online version contains supplementary material available at 10.1186/s12913-024-11117-6.

## Introduction and background

Parenting is an intricate process involving the upbringing and caring for a child from infancy to adulthood through promoting and supporting the child’s physical, emotional, social, and intellectual development [1]. This process becomes challenging, particularly when it involves preterm infants admitted to the hospital [2]. The birth of a preterm infant can be an epoch-making, evocative, and occasionally devastating parental experience [3]. A preterm infant is defined as a child born before the 37^th^ week of pregnancy is completed [4]. Annually, approximately 15 million preterm births are documented out of 160 million live births, accounting for an 11.5% global preterm birth rate [5]. Between 2010 and 2020, more than 60% of global preterm births occurred in South Asia and Sub-Saharan Africa [5]. One in every seven infants in South Africa was born before their due date and required NICU admission [5].

The NICU is typically a foreign and intimidating environment for parents, due to the need for continuous monitoring and medical intervention for infants who are fragile and sick. Parents can experience stress, guilt, anxiety, and sadness due to the infant's uncertain health prognosis [6]. The active involvement of parents in preterm infant care activities in the NICU is crucial for infant developmental outcomes [7]. Healthcare professionals should comprehend the parental experience of caring for a preterm infant in the NICU to address parental needs and enhance parent-infant interaction and attachment [8]. This interaction may in turn increase parental satisfaction, thus promoting more appropriate parent-infant interaction, including attachment and bonding [9].

Although parent-infant interaction is beneficial, cultural variables need to be acknowledged. Parenting is deeply rooted in a culture characterized by ideologies concerning how an individual should act, feel and think as an in-group member [10]. Therefore, the parental involvement and parent-infant interaction might be disrupted if the parental cultural practice is not considered. Cultural practices influence the parents' infant care approach [11, 12]. The values and ideals of culture are conveyed to the next generation through child-rearing practices, which implies that cultures are contextually sensitive parenting guidelines [13].

Parents of preterm infants in Limpopo Province, South Africa, come from various cultural backgrounds, which may influence how they understand and react to the care provided to their preterm infant in the NICU. Various childrearing practices associated with culture influence the health of preterm infants [14]. These practices include massaging the baby, applying oil to the eyes and ears, burping the baby, applying black carbon to the eyes, and trimming the nails. Parental involvement in preterm infant care in the NICU may also be influenced by culture [15]. The cultural views and ideas of healthcare professionals can potentially affect the standard of care offered to preterm infants and their parents in the NICU. These cultural views and ideas are health beliefs that explain the cause of illness, its prevention or treatment methods, and the appropriate individuals who should participate in the healing process [16].

Healthcare professionals who have a comprehensive understanding of the parental cultural determinants can facilitate the nurturing and promoting of adequate parental-infant care and interaction, which is the foundation for developing preterm infants [17]. Lack of support from healthcare professionals regarding the cultural aspects of parent-infant interaction may negate parents' cultural practices, and increase negative perceptions and dissatisfaction with the healthcare service provided in the NICU [17]. Consequently, this may result in a lack of parental awareness or responsiveness to the infant, associated with delayed infant cognitive development and multiple behavioural problems [18].

Despite the recognition of the importance of parental involvement in NICU care and the documented emotional challenges experienced by parents, there is a gap in the literature regarding the specific experiences and cultural practices of parents caring for preterm infants which can be integrated in NICU in settings, such as Limpopo Province in South Africa. The province has seen a significant increase in the number of newborn babies weighing under 2,500 grams in recent years [19]. The study aimed to explore and describe the cultural determinants of parents that can be integrated into the care of preterm infants admitted to the NICU in Limpopo Province to ensure culturally sensitive care. This study is unique due to its focus on South Africa, specifically Limpopo Province, which is the centre of cultural practices due to its rurality. The main research question was: *'What are the cultural determinants which influence the parental experience that can be integrated into the care of preterm infants in the NICU in Limpopo Province?*

## Methods

The Consolidated Criteria for Reporting Qualitative Research (COREQ) checklist was followed to ensure standardisation in reporting the study type, design, execution, analysis, and results [20].

### Design

The study applied a qualitative research design following a descriptive approach [21]. In-depth individual interviews were used to explore and describe the experiences of parents of preterm infants admitted to the NICU in Limpopo Province through a cultural determinant lens.

### Context

The current study was conducted in the NICU of a tertiary hospital in Limpopo Province, South Africa. Limpopo Province was selected based on two grounds: 1) Cultural practices are more evident in rural villages than in semi-urban or urban settings [22], 84.2% of the study population live in rural areas [23]; and 2) one in every seven infants is born before its due date in South Africa [5], the Limpopo Province accounts for high preterm birth rates [19].

### Participants and sample

The population comprised mothers of a preterm infant admitted to the NICU. For this study, a parent was defined as the mother of a preterm infant in the NICU. Purposive sampling was used to select twenty (*n*=20) participants from the NICU in a tertiary hospital [24]. The inclusion criteria required that 1) the participant be the parent of a preterm infant; 2) the parent had a preterm infant in the NICU for a minimum of two weeks (As set out by the researcher, the contextually relevant time for an immersive experience was two weeks); 3) the parent be able to speak either Sepedi, Xitsonga, Tshivenda, or English (the common local languages). Mothers of preterm infants who were in critical condition were excluded. There were no refusals to participate. The sample size was determined based on data saturation, which was reached with *n*=20 participants [25].

### Recruitment

The first author (Ph.D.) and an independent person recruited the participants face-to-face by distributing recruitment material such as flyers and asynchronously by displaying posters on the noticeboards in the selected hospital's NICU and a place where the mother lodge in the hospital. Recruitment was conducted after ethical approvals and permission from the hospital were granted. Participants who expressed interest in the study notified the first author through a phone call, SMS, or WhatsApp text message. The first author then contacted the potential participants to provide detailed information regarding the study aim and data collection method, including audio recordings of interviews, confidentiality agreements, written informed consent, and voluntary participation. Potential participants who showed interest were given an informed consent form and a minimum of 48 hours to consult and inform their partners or family members. The first author was accessible telephonically for any clarity-seeking questions. The first author contacted the agreed participants to schedule the hospital-based interviews on the agreed-upon dates. All consented mothers participated and there were no withdrawals.

### Data collection

The interview guide was developed for this current study in English, and translated to local languages (Sepedi, Xitsonga, and Tshivenda) by assistant researchers who are fluent with these respective languages. Three bilingual speakers (Sepedi, Xitsonga, and Tshivenda) checked the translations from English to these local languages for accuracy, which was endorsed. Furthermore, the interview schedule was piloted with two participants to assess its effectiveness and suitability (See supplementary document 1). Pilot study was instrumental in refining the interview guide and ensuring that it would yield the desired data during the primary study. The in-depth interview began with an open-ended question, as shown in Table 1 below.

**Table 1:** Interview guide

Question 1. Please tell me about your experience regarding parenting a preterm infant in the NICU
Question 2. What are the cultural/traditional/ritual/religious practices which influence your experience that can be integrated into the care of preterm infants in the NICU?

The data was collected between August and September 2022. In-depth individual interviews were conducted by the first author and assistant researcher using Sepedi, Xitsonga, Tshivenda, or English in a private room in the hospital to ensure confidentiality. COVID-19 precautionary measures were followed to protect the health and safety of participants and interviewers. Furniture was wiped with a 70% based-alcohol solution before and after each interview, chairs were spaced 1.5 meters apart to ensure adequate social distance and researchers and participants sanitised their hands before entering and exiting the room. Participants and the interviewer wore a surgical facial mask covering the nose and mouth throughout the interview.

The first author served as the lead interviewer, the assistant researcher functioned as a support system in case of a language barrier. The interviews were conducted in the participant's preferred language (Sepedi, Xitsonga, Tshivenda, or English). The interviewer used probing questions to encourage the participants to elaborate, and all other questions arose from the dialogue. The duration of each interview was between 45 and 65 minutes.

With the participants' permission, two audio recording devices were used to record each interview, whereby one served as a backup in case the main one defaulted. During each interview, the first author compiled field notes regarding the context, non-communication cues, and impressions to complement the recorded audio. Data collection continued until no new data emerged, whereby data saturation was declared. All the interviews were conducted at the hospital.

After data collection, the first author and assistant researchers transcribed the data verbatim, including field notes in English. The researchers' subjective experiences regarding the explored phenomenon were described to avoid influencing data analysis: a process termed bracketing [26]. Three bilingual speakers (Sepedi, Xitsonga, and Tshivenda) checked the translations to English transcriptions for accuracy against the audio recordings. Additionally, two transcripts (10% of the sample) were back translated, and accuracy was verified by an independent co-coder and two co-authors [SSM, KDS]. No substantial linguistic issues were identified during the translation process.

### Data analysis

Giorgi's data analysis method [27, 28] was applied to comprehend the essence of the experiences of parents of preterm infants in the NICU. The data analysis process constituted five steps: understanding raw data, constructing a constituent profile, forming a theme index, merging participants' theme indexes, and searching the thematic index to develop interpretive themes.

### Trustworthiness

The four criteria of Lincoln and Guba [29] were applied to establish the trustworthiness of the current study. Credibility was established by member checking with 10% of the sample (*n*=2) by sending the transcript and developed themes. The supervisors (experts) conducted a confirmability audit of the study project by checking and rechecking the collected raw-, coded- and interpreted data to affirm neutrality. Additionally, the study followed a rigorous descriptive qualitative method and underwent a peer review process that confirmed the consistency of the data, and the findings ensured dependability, while data saturation and a detailed description of the methodology ensured transferability.

## Results

### Demographic data

Twenty (*n*=20) mothers of preterm infants admitted to the NICU in a tertiary hospital participated in this study. The participants’ ages ranged from 18 to 39 years, with the majority being between 18 and 25. The majority of parents had three children. Regarding education, nine participants had a secondary-level education, and 11 had a tertiary education. Of the 20 participants, nine were unemployed, two were self-employed, one was fully employed, one was employed part-time, and seven were students (Refer to Table 2).

**Table 2:** Participants' demographic data

Participants	Age	Parity	Qualifications	Employment status
P01	18	1	Tertiary	Student
P02	25	2	High school	Unemployed
P03	26	3	Tertiary	Student
P04	24	2	High school	Unemployed
P05	38	5	Tertiary	Self-employed
P06	23	2	Tertiary	Student
P07	32	3	High school	Unemployed
P08	31	3	Tertiary	Unemployed
P09	30	3	Tertiary	Self-employed
P10	39	5	High School	Unemployed
P11	27	1	Tertiary	Unemployed
P12	28	3	High school	Fully employed
P13	22	2	Tertiary	Student
P14	39	6	High school	Part-time employment
P15	32	3	High School	Unemployed
P16	38	3	High School	Unemployed
P17	23	3	High School	Unemployed
P18	20	1	Tertiary	Student
P19	23	1	Tertiary	Student
P20	19	1	Tertiary	Student

### Emerging themes and sub-themes

Four main themes emerged from the data analysis. These were: lived experienced by parents of preterm infants, interactions with healthcare professionals, cultural practices concerning preterm infant care, and indigenous healthcare practices for preterm infants. These themes, supported by sub-themes, are outlined in Table 3.

**Table 3:** Summary of themes and sub-themes

**Themes**	**Sub-themes**
1. Lived experienced by parents of preterm infants	1.1 Stress and Exhaustion 1.2 Longing for home
2. Interactions with healthcare professionals	2.1 Care in NICU 2.2 Communication in NICU 2.3 Attitude of healthcare professionals in NICU
3. Cultural practice concerning preterm infant care	3.1 Infant naming by senior family members 3.2 Infant access restrictions 3.3 Family involvement 3.4 Religious practice observance/beliefs
4. Indigenous healthcare practices for preterm infants	4.1 Cultural practices used for cleaning the umbilical cord 4.2 Treatment of dehydration *“phogwana or lebalana*” 4.3 Care of eyes, ears, and nose 4.4 Infant bathing practices

### Theme 1 lived experienced by parents of preterm infants

The current study's first theme emerged as the lived experienced by parents of preterm infants. Parents experienced considerable challenges while caring for the preterm infants in the NICU. Lived difficulties experiences by parents are further explored through the sub-themes: stress and exhaustion, and longing for home.

#### Sub-theme 1.1 stress and exhaustion

Participants felt an overwhelming sense of exhaustion and stress, as they cared for their infants in the NICU. Participant responses revealed a pervasive fear of the unknown, coupled with emotional turmoil and physical strain. The uncertainty surrounding the health of their infants exacerbates their distress, leading to heightened anxiety and feelings of helplessness. This emotional burden is compounded by the challenges of navigating complex medical information and coping with unexpected health complications. Participants expressed shock, describing the unexpected event of preterm birth and the overwhelming emotions following the delivery of a preterm infant.

One participant reported:

*We are always scared when we go to see babies because we don't know what it is, especially when you leave the baby without the tube; you think she may vomit when you are not around, and the next thing you will be receiving a call saying your baby is no more.* (P1, 18-year-old)

Participant 1's expression of fear illustrates the constant apprehension experienced by mothers in the NICU, highlighting the emotional strain of anticipating potential emergencies and adverse outcomes. Another participant indicated the overwhelming uncertainty faced by the mothers upon entering the NICU, emphasizing the need for clear communication and reassurance from healthcare providers. The following quote supports the participant’s experience:

*What if they tell me the situation is like this when I enter there? Honestly speaking, it frightens us. We just wish that it didn't ring so that when you get there, they tell you that they needed you so and so*. (P2, 25-year-old)

Another participant highlighted the shock and fear induced by the sight of an extremely premature infant, illustrating the emotional toll of witnessing their vulnerability.

*This baby, she was too small, like it was the first time seeing a small child like this. I once saw premature, but it was not like this, this one was so small, so I was scared.* (P4, 24-year-old)

Another participant described her emotional response to distressing news about her baby's health which underscores the profound impact of medical uncertainties on maternal well-being, emphasizing the need for sensitive communication and support.

*When they told me that my baby was like this and this, I even cried.* (P10, 39-year-old)

Additionally, other participant’s narrative reflected the overwhelming fear and uncertainty experienced by mothers in the NICU, highlighting the emotional toll of constantly anticipating adverse outcomes and navigating complex medical situations. The following quotes reflect the participants’ experiences:

*What I'm dealing with, because I was very broken and did not know what it is, will the baby survive, what will she do, what's going to happen. The answer is not right, as, for us, we are always afraid, we don't know what it is when you are here.* (P17, 23-year-old)

#### Sub-theme 1.2 longing for home

The emotional strain and challenges faced by mothers while caring for their infants in the hospital setting evoke a profound yearning for the sense of security, comfort and belonging that home provides. The participants described their experience of not getting enough rest and sleep while caring for their preterm infant in the NICU which would not happen if they were home. Contributing factors include the time required to visit the NICU for the infant’s care routine, time spent walking from the mother's lodge to the NICU and back, and the separation of mother and infant.

The participant reflected on the contrast between hospital practices and what would have been done if she were at home:

*Yes, here in the hospital, they want you to bathe the baby like this while at home they want you to do this and this or at home, you would do this when you see him doing that. It's things I want to know.* (P5, 38-year-old)

This quote encapsulates the longing for the familiar routines and comforts of home amidst the unfamiliarity of hospital protocols. It highlights the sense of control and autonomy associated with home, where individuals adhere to their own customs and practices, as opposed to the regulated environment of the hospital. Another participant reminisces about cultural practices that would have been observed in her home environment:

*Yeah, like mostly, like back at home, in our culture, we believe that a baby less than a month old must be bathed by the mother or grandmother... If I was at home, I will be feeding her with soft porridge without giving her any medications because this medication makes her defecate twice a day or so and this makes her lose weight.* (P7, 32-year-old)

This excerpt emphasises the role of cultural traditions and familial support in shaping caregiving practices. It underlines the interconnectedness between home and cultural identity, where adherence to traditional customs provides a sense of security and belonging, particularly in the context of new-born care. Furthermore, another participant described the traditional approach to newborn care back home:

*No, after birth when I come home, we don’t bathe the baby right away, we dampen the cloth in lukewarm water and just wipe the baby where he is dirty. We wash the head because the hair traps a lot of dirty things (blood and birth secretions), we avoid the full bath so that we don’t expose the baby to flu.* (P18, 20-year-old)

Other participants compared hospital feeding methods with traditional practices at home. The participants reported that:

*Here we feed the baby with breast milk using pipes (NG tubes and syringes) but at home, we do a light and very soft porridge.* (P16, 38-year-old)

This comparison highlights the adaptation to different environments and the longing for familiar routines. It shows how home serves as a sanctuary where individuals adhere to their preferred methods of infant care, reinforcing the notion of home as a place of comfort and familiarity. Other participants expressed a longing for the comforts of home and the familiar routines:

*So, the first challenge is that we wake up. We only sleep two hours. Most of the time we spend on the way, we do not have time to rest. Like when you are going that way, you may find that you are going for a long time in the baby’s room. When you are coming here, and you try to sleep, time is gone, you must go back.* (P14, 39-year-old)

This statement reflects the desire for a sense of normalcy and routine amidst the challenges of hospitalisation. It highlights the idea that home represents a heaven of rest and recuperation, where individuals can adhere to their preferred practices and routines, particularly during significant life events such as childrearing.

### Theme 2 interactions with healthcare professionals

In this study, interaction is perceived as communication and involvement in preterm infant care among healthcare professionals and parents of preterm infants in the NICU. The sub-themes included NICU care, communication, and healthcare professional attitudes.

#### Sub-theme 2.1 care in NICU

This sub-theme concerns how healthcare professionals cared for preterm infants and their parents in the NICU. Some indicated that they received adequate care from nurses and doctors in the NICU.

One participant indicated that:

*The doctors are mostly here; they used to come only to check and update [us] about the baby's condition. The people who take part mostly are the nurses. Okay, looking at the ICU there is no problems, all is right.* (P02, 25-year-old)

A similar view was echoed by another participant who stated:

*Yes, they help me take care of the baby, and the doctors are nearby if there is something the doctor and nurses can help with.* (P13, 22-year-old)

Another participant shared that she had only seen good things and is at peace with the care that she is receiving in the NICU:

*In [N]ICU I have not seen any bad things; I only noticed the good things. My baby was in troubles, but she is not well, nurses are checking her every time so does the doctors. So up to so far, I never had any problems with nurses and doctors. I am at peace.* (P18, 20-year-old)

However, one participant expressed dissatisfaction with the care she received in the NICU. The following quote confirms this:

*They end up swearing at us and to be treated this way, been shouted, it ends up affecting our minds since I already have a problem with my baby’s condition.* (P12, 28-year-old)

The participants’ responses highlight that they experienced positive and negative care while looking after their preterm infants in NICU; it appears that they appreciated the care, although some were unhappy.

#### Sub-theme 2.2 communication in NICU

Nearly all parents mentioned the importance of healthcare professionals practising effective communication as clinicians. In this context, communication is the exchange of information between parents of preterm infants and healthcare professionals in the NICU. The parents indicated that they had experienced satisfactory communication with healthcare professionals while caring for their preterm infant in the NICU. This includes comprehensive explanations; for instance, the doctor offered information regarding the baby's weight decrease in terms that parents could comprehend, giving them relief. The following quotes support the experience:

*Yes, is not it that when we come here, we are under stress? So, if we want to say sister (nurse), may I ask, how is my baby doing? She can answer me; if she does not know, she must go and ask or tell me that I do not know about this one. I can ask someone who knows, like have good communication.* (P17, 23-year-old)

*Yes, the same doctor that I ask him regarding the baby’s weight loss. He explained to me well and now I understand, am free because the weight is no longer 0.8 kg, it is now around 1.0 kg. The support is good because when you ask something they quickly actioned it, so there is support.* (P18, 20-year-old)

The above participants highlighted the importance of efficient communication in interactions between parents and healthcare professionals in the NICU and its positive effects on parents’ experiences and well-being. Nevertheless, other participants expressed dissatisfaction with the communication they received from the healthcare professionals in the NICU. One of the cited reasons for their dissatisfaction was that healthcare professionals discussed the infant's condition in a language the parents did not understand.

One participant mentioned that:

*They asked me if I knew why my baby went to the theatre? What is the reason he came here? I said yes; I just heard them saying it is the authority which I do not know what they meant.* (P06, 23-year-old)

Similarly, other participants expressed disappointment that healthcare professionals were not informing them about the interventions/procedures before implementation. The following quotes support the parental disappointment:

*We do want to know because when we arrive in the ward, we just see that intravenous lines were inserted, and blood sample were collected, and we also see that the infant was pricked several times on the extremities hence do not even know where the samples are taken to.* (P16, 38-year-old)

*It is the same as when he was in high care because after labour, my baby was sent to high care, and the next morning he was in ICU without informing me.* (P19, 23-year-old)

Moreover, another participant mentioned feeling confused because of the conflicting communication from healthcare professionals. The following quote supports this confusion:

*The other one enters tells you the baby should change sides and give you reasons. When you tell them one said I should not change sides, they end up swearing at us end up confusing us.* (P12, 28-year-old)

Participants highlighted the negative impact that poor communication could have on their experience in the NICU, as well as the significance of simple and consistent communication with healthcare professionals. They expressed a desire for precise, reliable information to understand what was happening to their preterm infants and to feel more involved in the care of their infants.

#### Sub-theme 2.3 attitude of healthcare professionals in NICU

The participants in the study expressed dissatisfaction with the attitudes of healthcare professionals in the NICU, as illustrated by the two quotes below:

*When you go to the nurse and tell her that the tube is disconnected from the baby and the secretions are coming out through the nose, so the response will be like, what do you want me to do because your baby did that (mother rolling the eyes)?* (P01, 18-year-old)

*Okay there was this nurse who was on a night shift yesterday and she was busy with files, and when we wanted to ask her to collect some of the things for us, and she would say to us that we must go collect those things for ourselves because she is busy. So, when we got there to collect for ourselves, we found another nurse who asked us as to where our nurse is because we should not be doing this for ourselves. So, when we called her, she showed to me that she does not like her job.* (P08, 31-year-old)

Another participant further mentioned that:

*There is a nurse that seemed to have an advanced age, whenever we ask her to assist our babies, or asking some supplies to help our babies she is rude. She once told me that [my] babies are ugly such like me.* (P20, 19-year-old)

More so, some mothers lamented the lack of communication from the nurses. For example

*Their communication is not good because they hide things from us, sometimes you will find that they had taken your baby’s blood and not tell you about the results or what the results implies, and even when you ask the nurses, they would tell you that they are doing what they have been instructed to do. Sometimes you also find your baby inserted with drip, and when you ask, they do not say or explain the reason for all of these.* (P08, 31-year-old)

Even though other participants expressed their dissatisfaction regarding the attitude of the healthcare professionals, other participants felt the opposite. One participant mentioned that she had a satisfactory relationship with the healthcare professionals expressed in the quote:

*I am pleased with how the hospital is providing her with milk, yes, I am happy they help.* (P12, 28-year-old)

Similarly, another participant added that she has only observed good things concerning the level of service provided to her infant:

*In [N]ICU I haven’t seen any bad things; I only noticed the good things.* (P18, 20-year-old)

Most parents expressed satisfaction with the level of support provided by the healthcare professionals in the NICU. The participants describe the support as encouraging and helping them to understand that challenges are a normal part of the process, as indicated by the below quotes:

*Yes, their support is good. It is the kind of support that encourages you to understand that things like this are there and there are these kinds of challenges.* (P07, 32-year-old)

Additionally, another participant alluded that:

*The support from the nurses is very good, each one of them know me because I have been here for a long time. When they arrive, they call and ask how is the baby [doing]? Initially it was scary because my baby was the smallest one in the unit, and I was new but now am used to the nurses and the unit.* (P18, 20-year-old)

### Theme 3 cultural practice concerning preterm infant care

The third main theme was the cultural practices concerning preterm infant care. This study's concepts associated with this theme include practices and behaviours conducted after childbirth. This includes the infant naming practice, infant access restrictions, family involvement, and religious practice observance.

#### Sub-theme 3.1 infant naming by senior family members

Participants indicated that they adhere to the cultural practices of naming the preterm infant after birth. These cultural practices include understanding who is responsible for naming the infant, introducing the infant to the ancestors, and the meaning associated with the name given. The quotes show that senior family members, particularly grandmothers, are responsible for naming the infant and performing ancestral veneration to introduce the infant to the ancestors after birth.

One participant shared that:

*If the granny [was] still alive, she [would be] the right [person] to appoint my parent to name the infant.* (P02, 25-year-old)

Another participant supported the preceding statement by stating:

*Well, when I call them at home regarding the name, my grandmother would want her name to be passed down to the child.* (P08, 31-year-old)

The above data highlight that the grandmothers are responsible for naming the infants. This is because naming a preterm infant in Limpopo Province is culturally associated with the practice of ancestral communication, which grandmothers perform. Furthermore, one participant indicated that the infants are named based on various events in life. The following quotation illustrates this:

*Because they used a dead person's name, so they are informing the owner of the name that there is someone who will use it.* (P02, 25-year-old)

The above quote highlights the belief that a preterm infant is given the name of a deceased person to keep their memory alive and to ensure the continuation of a family legacy. Also, ancestral communication rituals should be performed to inform the name's owner. In addition, another participant indicated that infant naming is culturally essential and that a misnamed infant will continuously cry. The following quotation evidences this belief:

*They do that; for example, they can call a baby by name like Sara, and if the baby stops crying, it means that is the name she wanted. And these things happen because they can call her by her name; the baby then stops crying and is healed instantly.* (P01, 18-year-old)

The above data suggest that naming a preterm infant may positively affect the infant’s health and well-being when culturally informed. The beliefs and practices related to naming a preterm infant reflect the cultural values and traditions of the parents, which are essential considerations in providing culturally sensitive care in the NICU.

#### Sub-theme 3.2 infant access restrictions

Participants indicated that everyone is not permitted access to the room where the preterm infant is kept. Access restrictions include funeral attendees, pregnant women and individuals who recently engaged in sexual activities. The following section further explores how participants perceived these restricted individuals as harmful to the infant through a cultural lens based on their experiences during preterm infant care. A common experience for many participants was that individuals who participated in funeral services should perform cultural rituals with ashes and some aloes when entering, as illustrated in the following quotes.

One participant mentioned that:

*They [those attended the funeral] enter the baby's room, they bath the baby with aloe and ashes a little bit and even on the joints so that she must never get sick.* (P01, 18-year-old)

Another participant supported the preceding statement by stating:

*Usually, when they are from a funeral, they take ashes, apply them to the baby and make her swallow a bit of it so that they do not suppress her.* (P02, 25-year-old)

An additional participant concurred with the preceding participants and elaborated that:

*According to culture all babies from newborn to a child aged 6 to 7, when one person at home goes to the funeral, when that person comes back home takes ashes and rub it on the tummies of all these age group so that none of them can get suppressed or have negative auras.* (P20, 19-year-old)

The data highlight the cultural belief that there are diseases and negative auras that can be acquired from funeral services and that precautionary measures must be taken to prevent the spread of these harmful elements to the preterm infant. In addition to the precautionary measures highlighted above, other participants explained that people who attend funerals should be isolated from the infant for some period before regaining access to the infant's room, as illustrated by the two quotes below:

*I am staying with my grandmother, but if they are from the funeral, it means only I will nurse the baby. They will take seven days without entering the baby's room.* (P04, 24-year-old)

*She [person attended the funeral] must stay there for seven days before she returns, and after that, she can come back and help me with the baby.* (P11, 27-year-old)

The above quotes indicate that isolating individuals who attended the funeral service for seven days will allow the acquired diseases and negative auras from the funeral to clear up and minimize the chances of transmission to the infant. Pregnant women were the second restriction. The following quotes illustrate beliefs and practices surrounding the presence and interactions of preterm infants and pregnant women:

*Traditionally, we think she will suppress the baby. If a pregnant person carries the baby, she will delay the baby's growth. You find that at around six months, the baby is still unable to sit, so they believe it is because a pregnant person carried the baby. She is not supposed to enter the baby's room until the baby gets out.* (P02, 25-year-old)

Another participant said:

*If someone is pregnant, she is not supposed to hold a baby in such a way that the legs of the baby are on [her-pregnant woman] abdomen because we believe that if the baby's legs are stepping on top of the pregnant person's abdomen, the baby won't walk until the pregnant woman give birth, she will wait for the unborn baby to be born before she can walk.* (P01, 18-year-old)

The first quote highlights the complete restriction of pregnant women from gaining access to the infant due to the negative impact (slow growth) that she can have on the infant. However, the second quote indicated that a pregnant woman can be granted access to the infant’s room and can even carry the infant, although with precautions not to allow the infant’s leg to come in contact with the abdomen. Through this analysis, it becomes clear that cultural beliefs and practices play a substantial role in shaping the experiences of pregnant women and their interaction with preterm infants. The final restriction was holding the infant after sexual intercourse. Most participants revealed a common belief that sexual intercourse could lead to the transfer of a negative aura to the infant. The following quote exemplifies this belief:

*When the cord has not yet fallen, my grandmother is the only person who is allowed to enter because she has passed that stage of sexual intercourse. The rest of them are not allowed because we are trying to avoid negative aura to be passed on to the child, and if that happen, he will cry a lot. So, no one is allowed except my grandmother.* (P08, 31-year-old)

Other participants stated, in support of the preceding statement:

*They [siblings] might be coming from their partners and you would find that they were intimate in a way, so their energies will affect the baby negatively.* (P09, 30-year-old)

*Because they [grandmothers] do not have sexual intercourse anymore and they have experience. Culturally, it is believed that people who had sexual intercourse had negative aura.* (P16, 38-year-old)

The data suggest that the role of grandmothers in caring for preterm infants is essential and safe as they are free of negative energies due to their age, experience, and abstinence from sexual intercourse. Furthermore, the data highlights that individuals who engage in sexual intercourse bring negative auras to the baby and are, therefore, not allowed to be in close proximity to the newborn. This cultural practice aims to ensure the well-being and health of the preterm infant by avoiding contact with individuals who have recently engaged in sexual intercourse.

#### Sub-theme 3.3 family involvement

Cultural practices concerning preterm infant care restrict infant access and allow family members to assist in caring for the infant. The following quotations illustrate participants' experience regarding family involvement while caring for the infant.

One participant stated that:

*When I am here, the nurses help me, which is the same when you are at home. There is no difference.* (P11, 27-year-old)

Another participant expressed a similar view:

*It is very important because when you get help as a new mom you also get time to rest, in my family they would bathe the infant and massage you.* (P12, 28-year-old)

In support of the above participants, another participant added that:

*At home it is better because we have people who are assisting us, and we have time to rest* (P16, 38-year-old)

The conclusion that can be drawn from these findings is that the involvement of family members in caring for the infant enabled the mothers to rest rather than continuously caring for the infant alone, which may be exhausting.

#### Sub-theme 3.4 religious practices observance/beliefs

In context of this study, most parents were religious and observed religious practices in terms of prayer and using *ditaelo* (church prescriptions - the church practices believed to be effective in curing the patient and preventing misfortune). This is connected to the belief that their infants would be protected from illness and be healthy, parents would be strengthened, and healthcare professionals would be granted wisdom to care for the infants. Most parents prayed to God for their preterm infant to get better and be healed. The following quotes illustrate this:

*I just thought my baby is going to die but because God is present, I prayed I got baby boy. Now I thank God because of my faith and even the doctors had confidence that the baby will be okay.* (P18, 20-year-old)

*I pray every time I go to the ward for God to give her life and when I leave, I do not know what they will do to her, to not be affected when a lot of activities are done to her body.* (P02, 25-year-old) Furthermore, parents also prayed for themselves and drew strength from their spiritual anchor to overcome the challenges they experienced while caring for their preterm infant in the NICU.

One participant stated that:

*I have a way of overcoming my fears and sadness through prayer so that I can be able to receive strength*. (P11, 27-year-old)

Other participants also highlighted this. For example, one participant indicated that:

*When I am down, I pray for 2 minutes and ask God for strength. Then after, I feel okay.* (P02, 25-year-old)

Moreover, participants did not only pray for themselves and their infants but also for healthcare professionals to have wisdom while caring for their infants. The following quotes demonstrate this intercession:

*I believe that is the reason I prayed, because evil spirits can block the doctors view for them not see anything.* (P12, 28-year-old)

*Until now I just pray to God to give wisdom to doctors so that they treat my baby well then, I can go home.* (P18, 20-year-old)

Lastly, one participant believed that prayer is more effective when performed in person, in the presence of others, rather than done alone. The participant stated that:

*I prefer that when I pray, I must be there with two or more people because the prayer becomes more powerful when you are many.* (P11, 27-year-old)

These findings highlights that the communal aspect of religious practices is vital for some individuals and that they believe that the power of prayer is amplified when performed with others. The quotes in this analysis indicate that the participants view prayer as connecting with a higher power, seeking strength, wisdom, and healing for themselves and the preterm infant in their care. Another aspect of religious practice, observance/practice called *ditaelo*, was also used to protect their infants from evil spirits and heal them.

### Theme 4 indigenous healthcare practices for preterm infants

The final theme from the data analysis was “indigenous healthcare practices for preterm infants,” which parents described as the beliefs, knowledge, and habits about health passed down from generation to generation in a specific community. This theme is further explored through the following four subthemes.

#### Sub-theme 4.1 cultural practices used for cleaning the umbilical cord

Most participants believed that the indigenous care method for the umbilical cord is a vital cultural practice related to preterm infant care. Although the participants used surgical spirit in the NICU, they expressed the practices of using various herbal formulations that they would like to incorporate in the NICU during umbilical cord care. The following quotes reflect this.

*I take table salt with that powdered wood soot and apply it [umbilical cord] on the cord every time you bathe the baby until it dries.* (P10, 39-year-old)

*We took soil from termite mound, chickens’ manure and placed them there for it to fall.* (P12, 28-year-old)

Additionally, the same view was echoed by other participants, explaining that:

*The herbs will shrink the cord, which will eventually fall off. After that, they will give you herbs to spread over the cord area, which will help the cord to close from inside. I was using the ashes to mix with Vaseline, then spread the mixture over the cord.* (P15, 32-year-old)

*We clean the cord with surgical spirit. Then we also use the head from the ‘matches’ stick and mix with the mouse poo and crush it down until is a fine powder. Then we apply the fine powder on the cord area.* (P20, 19-year-old)

In addition to the various preferred herbal formulations, other participants mentioned that they apply breastmilk on the umbilical cord to increase the rate at which it dries. This is evident in the following quotes:

*We do a full bath after two days with warm water, then clean the cord with the spirit, and apply breast milk so that the cord can dry and fall fast.* (P18, 20-year-old)

*They say we pour breast milk on the cord, like basically the newborn baby we need to apply the breast milk when I wake up in the morning, on the belly button.* (P17, 23-year-old)

Furthermore, despite using surgical spirit in the NICU, participants were dissatisfied with its effectiveness. Most mothers felt that the delayed umbilical cord drying, and detachment were caused using surgical spirits.

One participant mentioned:

*The way of taking care of children here is different; for instance, the surgical spirit is not so effective in cleaning and making the cord dry. The cord would have fallen by now if I was home.* (P05, 38-year-old)

Another participant expressed that:

*With home remedies it takes up to three days but with the surgical spirit, it takes seven days. It is fast if you do it traditionally.* (P13, 22-year-old)

In support of the above participant, another participant further explained that:

*We are staying with elderly people at our homes, so immediately after the baby is born, we start by treating her umbilical cord, which, culturally or religion-wise, is much faster than what we use here at the hospital, because even here at the hospital, they treat the cord by spreading spirit on the cord, but it takes time.* (P14, 39-year-old)

#### Sub-theme 4.2 treatment of dehydration “*phogwana or lebalana*”

Some illnesses experienced by newborns are deemed to be not-for-hospital treatment but require indigenous healthcare practices or treatment. For example, dehydration is an indigenous childhood illness called *phogwana,* which traditionalists treat through herbal formulations. Other participants were concerned that their infant might suffer from *phogwana* while admitted to the NICU.

One participant mentioned that:

*Maybe if I do things the way I am used to doing on the baby, he might recover, or maybe the baby has phogwana, and the doctor thinks it is something they can treat.* (P06, 23-year-old)

The following participant echoed a similar notion in support:

*When the baby is sick with lebalana, you do not take the baby to the hospital because they do not know how to treat that. You take her to someone. In Tshivenda, we say when the baby has lebalana, they must cut, burn things that came out of it, and then come to the baby… then the baby heals at the same time.* (P17, 23-year-old)

Furthermore, participants shared that *phogwana* needs to be treated by a traditional healer or with traditional medicine. This is evident in the following quote.

*If the phogwana is not beating well, there is a traditional medicine that we apply to make sure that it does not affect the baby.* (P16, 38-year-old)

#### Sub-theme 4.3 care of eyes, ears, and nose

The subtheme of *"care of eyes, ears, and nose*" within the major theme of indigenous healthcare practices for preterm infants is represented by traditional methods of addressing issues related to the eyes, ears, and nose. Most participants reported using breast milk to clean and treat minor ailments of the eyes, ears, and nose.

One participant shared that:

*Most of the time we use breast milk to take care of their eyes, and that even allows them to sleep peacefully, we take few drops of our breast milk and pour them inside his eyes.* (P08, 31-year-old)

Another participant added that:

*If the eyes are having discharge, we express breast milk inside the eyes and wipes it using the tongue to remove the discharges.* (P16, 38-year-old)

In addition to using breast milk for eyes, it was reported to treat blocked nostrils and common flu and clean the umbilical cord, as reflected in the following quotes.

*Breast milk works especially when the eyes are white or having the discharges. Same as the nose, when the baby is having a flu, we put few drops of breast milk that is our culture.* (P18, 20-year-old)

Participants believe that the non-nutritional use of breast milk as a remedy or treatment for minor ailments of the eyes, sinuses, and ears is effective. This traditional belief may be because breast milk contains antibacterial and anti-inflammatory properties.

#### Sub-theme 4.4 infant bathing practices

The current study further revealed that preterm infant bathing was not only done for hygiene-related reasons but was also seen as serving to stimulate weight, for physical strengthening, and to protect the infant against evil spirits. These reasons are reflected in the following quotes.

One participant indicated that:

*Traditionally we bathe her with sehlapišo (traditional medicine) used to bathe infants to stimulate weight gain.* (P13, 22-year-old)

Another participant also shared that:

*We use leaves from the Baobab tree to bathe the baby; it is a medication. It is responsible for making the baby strong.* (P10, 39-year-old)

In addition to herbal medicines that stimulate infant weight, other participants reported using herbal formulations to protect the infant against evil spirits and negative auras. The usage of herbal formulations is evident in the following quotes:

*They use mogato (a traditional form of medicine to protect the baby from being suppressed) for bathing her. They put mogato inside the water and then just bath her, more especially if there is someone from the extended family coming to visit.* (P02, 25-year-old)

*I use the mixture, add it to the water, and bathe the baby to remove the negative spirits and aura, and some is for weight-gaining stimulation because young babies are difficult to hold due to their size.* (P14, 39-year-old)

Lastly, other participants also shared the same notion; however, they indicated that this kind of herbal formulation called *sehlapišo* should not be used on the infant’s head during bathing as it is believed that the infant’s head will grow at an expedited rate should it come in contact with *sehlapišo*. The following quotes demonstrate this point:

*When we use ‘sehlapišo’ for two days, we keep the water and then the next day we dilute it with hot water so that it becomes warm, and then after bathing we rinse him. we only bath his arms and legs because if we bath his head and neck they will grow too as this is used for growing or gaining weight.* (P08, 31-year-old)

*You do not touch the baby’s head when using ‘sehlapišo’ you only bath him from the neck to his toes, because they say if it happens that you touch the baby’s head while bathing him, [otherwise] the head and face becomes swollen and changes size.* (P09, 30-year-old)

## Discussion

This study highlights parents' experiences caring for their preterm infants and the cultural determinants that can be integrated into preterm care to ensure culturally sensitive care. Four major themes and related sub-themes emphasise the importance of healthcare professionals respecting and acknowledging cultural practices, beliefs, and customs relevant to parents of preterm infants admitted to their facilities.

Participants in the current study experienced a range of negative feelings, including shock, fear, and anxiety, concerning the unexpected event of preterm birth, consistent with the literature. For instance, studies conducted in Sweden [30] and Denmark [31] reported that the abruptness of preterm birth, combined with the physical environment of the NICU, evokes feelings of shock and overwhelm in parents. Furthermore, the fear and anxiety experienced by the participants in this study while caring for their preterm infant in the NICU corroborate the findings in existing literature [32, 33]. Both studies reported that parents often oscillate between hope and fear, particularly regarding their infant's survival and the possible long-term health complications associated with preterm birth. This correlation could be explained by the fact that preterm birth is traumatic and a potential stressor because it occurs mostly under emergency conditions, often threatening both the parents and the infant's well-being.

The current study's findings revealed that most participants acknowledged receiving satisfactory care from the nurses and doctors, as they were regularly present and helpful in tending to the infants' needs. This finding mirrors those of a study which noted that parents appreciate the quality of care provided by healthcare professionals in the NICU [34]. However, some participants felt that the nurses were often not friendly and mistreated them in the NICU. The findings are similar to the study which reported that some parents were dissatisfied with the care they received, which often stemmed from perceived rude behavior or negligence [35]. While technical, medical treatment and care are vital, the current data highlight how such care significantly influences parents' experiences in the NICU.

Communication, both in content and manner, is essential in the NICU setting, as it profoundly impacts parental experiences [36]. In addition, communication was also identified as a critical component in providing quality care to a diverse population concerning incorporating culturally competent care [37]. The current findings showed that many parents were satisfied with the communication they received from healthcare professionals, particularly when they were given clear explanations about their infants' condition. However, specific communication issues, including using incomprehensible medical jargon, insufficient intervention information, and conflicting advice from different professionals, were pointed out. These issues align with previous research, highlighting the need for improved communication strategies in the NICU to better inform and support parents [38].

Regarding the attitude of healthcare professionals, our findings revealed a mixed perception among parents. Some parents expressed dissatisfaction with the perceived negative attitudes of healthcare professionals, echoing similar findings by Shields et al. [39]. Negative attitudes from healthcare professionals can lead to mistrust and increased stress among parents [40]. Conversely, other parents in our study reported positive attitudes and felt well-supported and valued by the NICU staff. This positive perception aligns with the previous study which suggested that positive interactions with healthcare professionals can improve parental satisfaction [41]. While the current findings corroborate existing literature, the heightened perception of both positive and negative aspects of care, communication, and attitude might be attributed to cultural diversity in Limpopo Province.

The current study found that naming preterm infants is the domain of senior family members, particularly grandmothers. This finding aligns with previous work which asserted that grandmothers play a crucial role in naming infants and performing associated rituals in African cultures [42]. This role could be because the naming process is closely related to ancestral communication, which grandmothers frequently facilitate. Furthermore, the study indicates that infants' names often carry important cultural meanings or memorials, reflecting events or individuals in the family's history. The belief in the power of naming to affect an infant's well-being corroborates with the previous study’s assertion that names in most African cultures bear profound significance, carrying the family's hopes, aspirations, and legacies [43]. Additionally, names help individuals understand who they are and the community to which they belong. Such findings underscore the importance of cultural considerations concerning naming preterm infants in the NICU to promote culturally sensitive care and enhance parents' experiences.

In this study, three cultural restrictions on infant access aimed at safeguarding preterm infants' health were revealed. These restrictions primarily concern those who attended funerals, pregnant women, and people who recently engaged in sexual intercourse. First, funeral attendants: participants believed they could introduce diseases or negative auras to preterm infants, so precautionary measures needed to be taken before access could be granted again. The precautionary measure, which includes isolating funeral attendants for several days and having them wash their hands with aloe and ashes before touching the infant, aligns with a study by McAdoo [44], which reported similar customs among various African cultures. The use of aloe and ashes might stem from the fact that they contain some antibacterial properties, which may kill or lessen bacteria.

Second, according to our findings, pregnant women were also viewed as potentially harmful to preterm infants. This finding is unique as no other similar study could be located regarding the harm that could be brought by pregnant women. Third, individuals who recently engaged in sexual intercourse were deemed to have negative auras that could harm infants, particularly from parents' perspectives. This restriction echoes findings of previous study which revealed that newborns are isolated from young girls who engage in sexual activities as they can delay umbilical cord falling off [45]. This finding highlights the need for open dialogue and understanding regarding sexual practices in NICU care.

This study's findings underline the key role of family members in caring for preterm infants, which aligns with previous research in the field. Particularly, participant responses corroborated the evidence of family involvement as crucial to maternal well-being and infant care, as shown in a study conducted in the United States [46]. The responses reflect an appreciation for the support offered by extended family, primarily in providing mothers with rest and recovery time, mirroring previous findings [47]. The significance of family engagement in this study can be linked to cultural norms and values in the Limpopo Province and South Africa.

Most South African tribes, particularly indigenous ones, strongly believe in communal assistance and interdependence, particularly at significant life events such as childbirth. This is frequently characterised by extended family members stepping in to aid and support the new mother, allowing her time to relax and heal while contributing to the infant's care. Additionally, the similarity in support between NICU nurses and family members emphasized by participants resonates with the notion of family-centred care advocated by other scholars [48]. This approach, which suggests that healthcare providers can emulate a sense of familial support, highlights the importance of aligning clinical practices with the socio-cultural context of care.

Most participants expressed a reliance on prayer for the health of their infants, personal strength, and wisdom for healthcare professionals, which aligns with other studies that demonstrated the importance of spiritual beliefs in health outcomes and coping mechanisms [49–51]. Moreover, the idea of communal prayer being more potent than individual prayer, as pointed out by one participant, echoes classic sociological theory on the collective effervescence and emotional energy generated in communal religious rituals [52]. This finding accentuates the importance of understanding and integrating spiritual needs and beliefs in the NICU environment.

Interestingly, participants in the current study also invoked '*ditaelo*', or church prescriptions, in protecting and healing their infants. This practice, not extensively documented in the existing literature, appears to be a distinct element of religious observance in this cultural context. It may relate to African traditional healing practices, as discussed in the previous studies which indicated a unique fusion of Christianity and indigenous beliefs [53, 54]. This practice underscores the cultural and spiritual complexity surrounding NICU care in the Limpopo Province and calls for further research to better comprehend these practices and their implications for infant care.

The participants’ experiences in the current study regarding umbilical cord care revealed that most parents reported using and believing in traditional cord care practices. These participants further described using ashes, powdered wood soot, breast milk, and soil from termite mounds topically to dry off and heal the umbilical cord. The use of herbs to treat and care for the umbilical cord was not unique to the participants in this study. In Sub-Saharan countries including South Africa [45], Zambia [55], Nigeria [56], Pakistan [57], and Uganda [58], the topical application of substances to the umbilical cord to hasten its detachment has been reported. It is important to acknowledge that while these traditional practices hold cultural significance and have been used for generations, their efficacy and safety may differ. In some cases, such practices may carry risks, such as infection or irritation. Healthcare providers should be aware of these cultural practices and engage in open and respectful conversation with families to understand their beliefs and preferences while also providing safe evidence-based care.

Moreover, participants also expressed dissatisfaction with modern procedures, such as surgical spirits, which they perceived as less effective than traditional practices because it makes the cord detach after seven days. This perception echoes the findings of study which revealed that some cultures believe traditional practices provide superior results compared to modern medical care, particularly for infants [59]. Although the herbal formulation was preferred over modern medical care, it has not been scientifically evaluated and studied; therefore, there is a potential risk of infection and other complications. Further research is needed to understand the scientific functionality of herbal formulations used to treat and dry off the umbilical cord.

This study showed that there are perceptions that certain medical conditions affecting newborns do not necessitate hospital care but rather require indigenous healthcare practices or treatment. For instance, *phogwana* was mentioned as a condition that needs out-of-hospital treatment by traditionalists. Similarly, this finding supports the previous literature which documented that the treatment of *phogwana* requires a traditional healer [44, 60]. In addition, the literature indicated that the characteristics, prevention, and treatment of *phogwana* correspond to specific cultural contexts [61]. Providing medical care for premature infants outside of the hospital, under the guidance of traditionalists, may pose result risks, such as adverse responses to herbal therapy and metabolic poisoning. The immature organs of preterm newborns may have limited ability to efficiently remove metabolites of herbal medicines, which could potentially cause more health complications and death [62].

Furthermore, regarding the care of eyes, ears, and nose, participants reported using breast milk as a treatment for minor ailments. The belief in the antibacterial effects and healing properties of breast milk in traditional medicine is further substantiated by this finding, aligning with existing literature. These studies reinforce the multifunctional uses of breast milk beyond nutrition, including its application in treating eye infections [63] and alleviating nasal congestion, among others [64]. Although the benefits of breast milk are recognised, it is crucial to follow proper hygiene protocols when dealing with it. This includes washing your hands before handling breast milk and using sterile containers and applicators. Neglecting to maintain good hygiene can potentially introduce infections to the ears, nose, and eyes.

The participants in the current study reported that infant bathing was performed with different herbs for several purposes, such as stimulation of weight, warding off the evil spirit, and strengthening and protecting the infant. Herbal formulations used for bathing included *sehlapišo*, *mogato*, and baobab tree leaves. This study's findings agree with several studies on the African continent. In Uganda, infants were bathed with *kyogero* to attract fortunes [65], and in South Africa [44], India [66] and Nigeria [67], herbal medicine was also used during infant bathing for strengthening and spiritual protection purposes. One possible reason for the similarity could be that all studies reporting indigenous infant bathing were conducted on the African continent, which has overlapping cultural practices. It is clear from this finding that bathing practices are not merely physiologically functional but are often symbolic, serving various socio-cultural purposes and highlighting the intersection of cultural belief and healthcare. Preterm infants are vulnerable to health risks such as hypothermia, skin irritation, and infection due to their underdeveloped thermoregulatory system, delicate skin, and immature immune system [68]. Ritual bathing, particularly if not performed carefully, has the potential to worsen these health risks. It is recommended that healthcare professionals should ensures measures to guarantee that the ritual bathing environment for preterm newborns is secure, hygienic, and at a suitable temperature to reduce these dangers.

## Limitations and strengths of the study

This study explored the cultural determinants of parents that can be incorporated into preterm infant care to ensure culturally sensitive care as part of maternal and childcare routine in the NICU in Limpopo Province. Although the qualitative design was the most appropriate to explore the phenomenon in this study, it limited the study's findings as it was not generalizable. Additionally, the primary investigator’s unconscious biases and perceptions could have influenced data analysis, however bracketing was applied to limit bias. Furthermore, to limit biases, the experts conducted a confirmability audit of the study project by checking and rechecking the collected raw-, coded- and interpreted data. The current study was conducted in a public hospital in Limpopo Province to explore the experiences of parents of preterm infants in the NICU, which may differ substantially from those in private hospitals and other provinces. Therefore, future research is recommended to explore this phenomenon in private hospitals and other provinces in South Africa.

## Conclusion

The current study provides an understanding of parents' experiences caring for preterm infants in the NICU. The study offered meaningful insights into indigenous healthcare practices, emphasizing their crucial role in preterm infant care in specific cultural contexts. The cultural determinants included various topics, such as caring for the umbilical cord, treating *phogwana*, caring for the eyes, ears, and nose, and infant bathing customs. These practices showed a deeply ingrained belief system and a rich cultural heritage that have a meaningful impact on healthcare behaviours. However, these cultural determinants might have both positive and negative implications.

The findings demonstrated a strong reliance on traditional methods and herbal formulations in caring for preterm infants. Parents emphasised the advantages of these practices over current medical procedures, notably in treating disorders not frequently recognised by modern medicine and the care of the umbilical cord. This discontent with contemporary practices, highlights the need for culturally sensitive healthcare which can be conducted by conducting cultural assessments to understand the beliefs, values, and practices of the families in the NICU.

Overall, the findings of this study highlight the profound role of indigenous healthcare practices for preterm infants, reinforcing the need for a culturally sensitive approach in healthcare.

### Supplementary Information

Supplementary Material 1. 

## Data Availability

The dataset materials generated and analysed during this study are accessible upon justified request from the corresponding author [MN].

## Abbreviations

NICU: Neonatal Intensive Care Unit
NWU: North-West University
