# Supplementary material for: Parental experiences of caring for preterm infants in the neonatal intensive care unit, Limpopo Province: a descriptive qualitative study exploring the cultural determinants
Source: BMC Health Serv Res. 2024 May 28;24:669. doi: 10.1186/s12913-024-11117-6 (PMC11134925; doi:10.1186/s12913-024-11117-6)
Supplement: Supplementary file 1 — Supplementary Material 1. [file 12913_2024_11117_MOESM1_ESM.docx]

ONE-TO-ONE face INTERVIEW schedulE

**Researcher’s role during one-to-one face interviews**

A private venue/room was selected and used at the hospital to ensure that all participants feel comfortable. The room was noise-free, well ventilated, and with comfortable chairs placed 1.5m apart. The researcher supplied two voice recorders, extra batteries, notebook, pen, and a 70% alcohol-based hand sanitiser. No refreshment was served.

# Interviews questions

Question 1. Please tell me about your experience regarding parenting a preterm infant in the NICU.

Question 2. What are the cultural/traditional/ritual/religious practices which influence your experience that can be integrated into the care of preterm infants in the NICU?

**Schedule**

- Firstly, the interviewer introduced himself
- The interview procedure was explained, including utilisation of the voice recorder.
- Questions that participants have were addressed by the interviewer.
- The interview began only after permission was sought.
- Prepared voice recorder and checked the sound quality.
- Initiated interview when the participant was ready.
- Informed the participants about research topic
- Reassured the participants that all the information is confidential and will be kept on a password encrypted device/computer.
- Made participants are aware that participation is voluntarily, and they can withdraw anytime, without any coercion or penalty.
- Field notes were taken during the interview by the interviewer, to note the situational context and non-communication cues and impressions.
- After asking and discussing all the questions, and when no new information was emerging, the interviews was terminated.
- Conveyed gratitude to participants for their time and doing the interview.
- Reemphasised and reassured participants of confidentiality.
- Any questions aroused from the participants, was answered.
- Discussed the data analysis process, sharing of findings was discussed as well as the article(s) to be published.
- Shared contact details with the participants should they want to contact the researcher regarding the research.
- Thanked participant.
